# Accuracy Investigation of the Pose Determination of a VR System

**DOI:** 10.3390/s21051622

**Published:** 2021-02-25

**Authors:** Peter Bauer, Werner Lienhart, Samuel Jost

**Affiliations:** Institute of Engineering Geodesy and Measurement Systems, Graz University of Technology, 8010 Graz, Austria; werner.lienhart@tugraz.at (W.L.); samuel.jost@tugraz.at (S.J.)

**Keywords:** virtual reality, accuracy, HTC Vive Pro, laboratory investigations

## Abstract

The usage of VR gear in mixed reality applications demands a high position and orientation accuracy of all devices to achieve a satisfying user experience. This paper investigates the system behaviour of the VR system HTC Vive Pro at a testing facility that is designed for the calibration of highly accurate positioning instruments like geodetic total stations, tilt sensors, geodetic gyroscopes or industrial laser scanners. Although the experiments show a high reproducibility of the position readings within a few millimetres, the VR system has systematic effects with magnitudes of several centimetres. A tilt of about 0.4° of the reference plane with respect to the horizontal plane was detected. Moreover, our results demonstrate that the tracking algorithm faces problems when several lighthouses are used.

## 1. Introduction

Virtual Reality (VR) has become a state-of-the-art tool for visualisation, gaming and education in virtual environments [1]. The integration of external objects into a VR application is a common way to realise a more realistic user experience. The usage of a car interior in a driving simulation or the inclusion of a table’s surface as a game board are widely used examples. Whenever real objects interact with the simulation, the accuracy of the position and the orientation (pose) of the VR system’s components in the real-world environment are crucial.

For the pose estimation of VR systems, different tracking approaches are used. These tracking approaches differ from their possible use cases, because some are designed for a quick and user-friendly setup and some are designed for a stable and accurate simulation. However, in both cases manufacturers of VR systems usually do not provide information about the achievable pose accuracy of their devices. This also applies to the HTC Vive system where the majority of the accessible information originates from user reports in web forums and video channels, e.g., YouTube. Due to the concept of the tracking approach and the user feedback, a high accuracy in the pose estimation is assumed for this HTC Vive system. Therefore, it is commonly used in various technical applications, for instance, for motion tracking in a robot control system [2]. A first developer inspection of the lighthouse system was carried out by Kreylos in 2016 [3]. His investigations focused on the update rate and drifts of the HTC Vive system and covered small scale accuracy investigation. In the report, the position measurements of the HTC Vive have been compared with a 36 inch ruler. Niehorster [4] has published the first scientific inspection of the lighthouse system of the first generation in 2017. The reference grid was set up with a spirit level and the WorldViz PPT-X device as reference system. A tilted horizontal reference plane has been detected and horizontal deviations of a few centimetres were reported. However, a detailed investigation of the behaviour of the deviation vectors was missing and the experiments have not been repeated with different lighthouse constellations. Moreover, a PhD thesis in 2018 has obtained comparable results with deviations of more than one centimetre with regard to a research grade motion capture device [5]. In the same year, NASA [6] presented an improved tracking algorithm for the first generation of the HTC Vive system using the raw data of the VR system. However, end users usually do not have access to this raw data and thus all investigations shown in this article use positions and orientations which are provided by Valve’s SteamVR software.

Since 2018, the second generation of the lighthouse system has been available, which originally supported only the HTC Vive Pro system. In the following year, Valve released its own VR System called the Valve Index. Both state-of-the-art systems use the same lighthouse infrastructure and the same SteamVR software for position and orientation estimation and distribution to third-party software [7]. The working principle of the second generation differs significantly from the working principle of the first generation which was officially presented by Alan Yates in 2016 [8]. Information on the working principle and accuracy of the lighthouses 2.0 originate mainly from unofficial online sources such as CNLohr [9] or Bezmalinovic [10].

In 2019, a team of researchers around van der Veen investigated the position accuracy of the HTC Vive Tracker in a dynamic 3D setup [11]. In their test setup, the tracker was placed on a movable robot arm. The investigations with the lighthouses of the second generation have shown in this case higher deviations than Niehorster [4] has published for his static investigations of the first generation. A final conclusion cannot be drawn as different hardware and test setups were used.

The concept of the lighthouse system is comparable to the iGPS system of Metris which is used in industrial metrology [12]. A capability study performed by the National Physical Laboratory has proven an accuracy of 0.1 mm position error for the iGPS system in a 10 m × 10 m test setup [13]. Therefore, it seems still promising that the HTC Vive Pro system is capable of a high accuracy with different restrictions even though it is low cost sensor.

This paper is intended to provide detailed information for the lighthouse system of the second generation and to determine the achievable position accuracy under controlled laboratory conditions.

A summary of acronyms and notations is listed in Table 1.

## 2. System Components of the Htc Vive Pro

**Figure 1:**
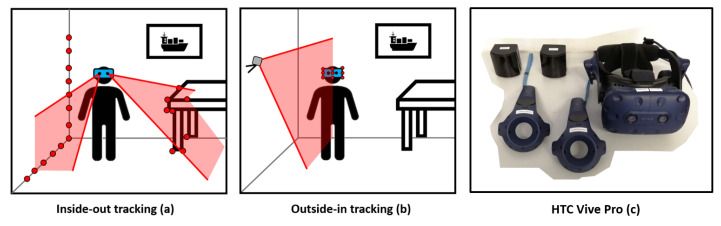
Different tracking procedures (**a**,**b**) and system components (**c**).

The HTC Vive Pro system (HTC Cooperation, Xindian, Taiwan) is a 3D indoor positioning system with a designated working range up to 7 m and, based on the available literature, errors of more than one centimetre can be assumed for the position tracking. The VR System HTC Vive Pro is sold with a Head-Mounted Display (HMD), two controllers and two base stations (Figure 1c). These base stations (Lighthouses 2.0, HTC Cooperation, Xindian, Taiwan) are part of the outside-in tracking procedure (Figure 1b). The lighthouses of the HTC Vive emit IR signals which are detected by IR diodes on each device. Valve has published that it is necessary that at least five diodes have to be observed by the lighthouse to estimate the position and the orientation of a device [14]. Other indoor positioning systems following an outside-in tracking procedure are for instance Bluetooth fingerprinting [15], High sensitivity GNSS [16], the iGPS system from Metris [12], or the CLIPS system of ETH Zurich [17].

In contrast to the outside-in tracking is the inside-out tracking procedure (Figure 1a), where the positioning system is independent from external reference signals as used in the HTC Vive Cosmos [18] or the Microsoft HoloLens [19].

The HTC Vive Pro HMD has a configuration of 31 IR diodes and the controllers of 24 IR diodes. Using the known configuration of the diodes, the time when a diode is hit by the signal and the time differences between the hits of the diodes, SteamVR can calculate all 6 degrees of freedom for each device. The lighthouse system can be operated with up to four lighthouses at the same time [7]. The Vive Tracker (HTC Cooperation, Xindian, Taiwan) from 2018 is an additional device to include external objects into a VR application. The Tracker has a known origin which is defined by the camera screw thread on the ground plane. With its 22 IR diodes, the Tracker uses the same positioning system as the HMD and the controllers. The camera screw thread enables the tracker to be mounted on an arbitrary object and to easily track that object’s position and orientation within the VR application [20].

## 3. Testing Environment

A representative investigation of the system behaviour requires a test setup, which is capable of sustaining a three dimensional reference with constant superior accuracy for the whole working range. Regarding developer studies like Kreylos [3], the accuracy investigations have been carried out on a very small scale and were aimed to detect the minimal position resolution of the HMD. The results published by Niehorster [4] have been the basis for all upcoming research, although he has used only a 2D reference grid and it is questionable if the visual reference system provided an adequate accuracy level.

In the publication by van der Veen [11], the lighthouse system has been used in a typical setup with two lighthouses in a dynamic test setup. Due to the high influence of the dynamic tracking algorithm of SteamVR in this test setup, these results are representative for a dynamic use case but may underestimate the reachable accuracy level of the Vive Tracker itself.

All of the investigations presented in this article have been carried out under controlled environmental and lighting conditions in the measurement laboratory of the Institute of Engineering Geodesy and Measurement Systems (IGMS) of Graz University of Technology (TUG). To minimize the effects of the dynamic tracking procedure of SteamVR a static test setup has been chosen for the investigations. The experiments have been carried out in setups with one and two lighthouses in different constellations to reveal possible systematic effects.

**Figure 2:**
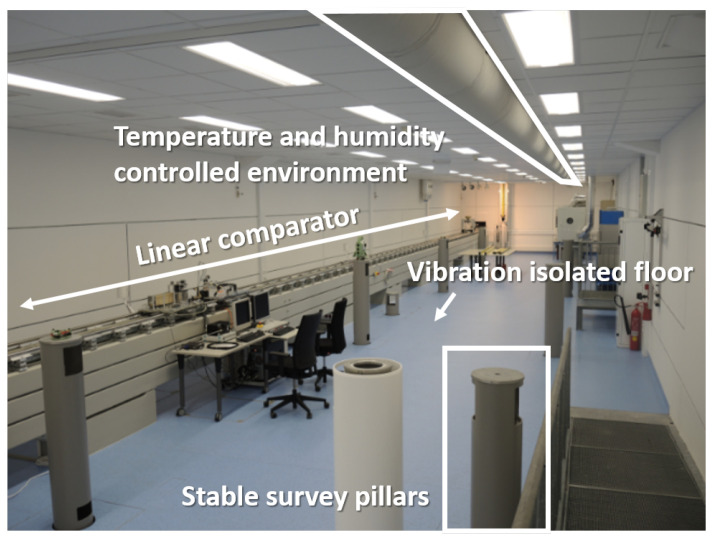
IGMS measurement laboratory.

The laboratory is fully air-conditioned (temperature 20 ± 0.5 °C) and has a vibration-isolated floor. Furthermore, it is equipped with stable surveying pillars for setting up total stations and creating a 3D reference system with sub-millimetre accuracy (Figure 2) throughout the whole working range of the lighthouse system. With this reference frame the position of objects can be determined with sub-millimetre accuracy as used in Section 6.1. In addition, the pillars can be used as a stable platform for rotation tests as performed in Section 6.4.

One of the main test infrastructure is a 25 m long linear rail system. This horizontal comparator is levelled with a height accuracy of better than 2 mm along its entire length and has an automatically movable carriage on it (test setup in Section 6.3). The position of the carriage along the rail can be determined by an interferometer with micrometre resolution.

This test facility is normally used for the calibration of opto electronical distance metres and for accuracy investigations of terrestrial laser scanners [21] and fibre optical sensors [22].

## 4. Statistical Analysis

The acquired data sets have been investigated on the basis of their systematic and stochastic behaviour. At each experiment an adequate amount of measurements has been recorded to describe the precision (or noise) and to perform a statistical test with the corresponding distribution (theoretical variance = Gaussian distribution, empirical variance = Student t distribution). The precision (noise) is described with the standard deviation of coordinate differences in the corresponding axis.

For statistical tests with a complex functional relationship the laws of variance propagation have been applied. All statements in the paper that have been made on the basis of a statistical test are marked with the term “significant”. All results in the paper have been tested for statistical significance on a confidence level of 95% (*p* = 0.95) which is commonly used in engineering geodesy [23].

The pose data of HTC Vive Pro devices has been recorded via the SteamVR Plugin 2.0 (Valve Cooperation, Bellevue, WA, USA) in a custom Unity application. All mathematical computations have been executed within the Matlab environment (version R2015b, Mathworks, Natick, MA, USA).

## 5. Precision of the VR System

The precision of a 3D positioning system can be described by the reproducibility of 3D positions at different time epochs. Reproducibility is a relative parameter and is not affected by systematic effects. Therefore, precision describes the noise level that is inherent in every observation.

### 5.1. Precision of the HTC Vive Pro Devices

**Figure 3:**
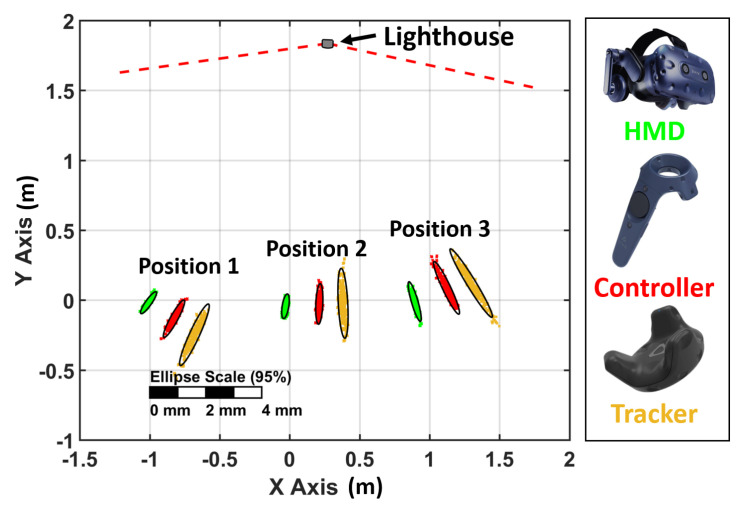
Noise pattern of all devices at three different positions with one lighthouse. Distance to lighthouse: 1.8 m. Individual position results (coloured dots), 95% confidence ellipses (black).

To investigate the precision of the HTC Vive Pro devices, 500 static 3D positions have been recorded with every device at three different locations. In this setup, only one lighthouse has been used to avoid any kind of compensation by redundancy in the position tracking. The resulting horizontal positions are plotted in Figure 3 and show an elliptic-shaped noise pattern for all devices.

This pattern is orientated towards the active lighthouse. This indicates that the noise in the positions is higher in the direction towards the lighthouse than orthogonal to it.

**Figure 4:**
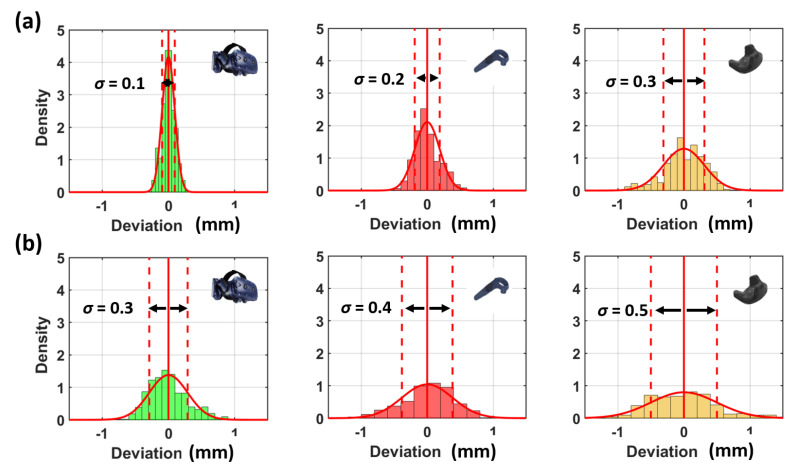
Histograms of the horizontal deviations (**a**) orthogonal (**b**) in direction to the lighthouse at position 2 seen in Figure 3. Standard deviation (σ) values are in the unit mm. Distance to lighthouse 1.8 m.

Therefore, the distribution of the noise pattern was further investigated in these two distinctive directions. As an example, the histograms of position 2 are displayed in Figure 4. The histograms are scaled according to the density functions and show a stochastic behaviour without any systematic effects.

The numerical values, which are displayed in Table 2, show that the magnitudes of the noise patterns correlate to the amount of IR diodes on the devices. Therefore, the VR Tracker has a higher noise compared to the controllers and the HMD in this setup.

### 5.2. Detailed Precision Investigation of the VR Tracker

Due to the similar behaviour of all devices and the importance of the Vive Tracker for mixed reality applications, it is the Vive Tracker that is examined in more detail. Valve recommends the use of at least two lighthouses in a regular setup [24]. This should improve the visibility of IR diodes and should lead to an improvement in the position tracking.

**Figure 5:**
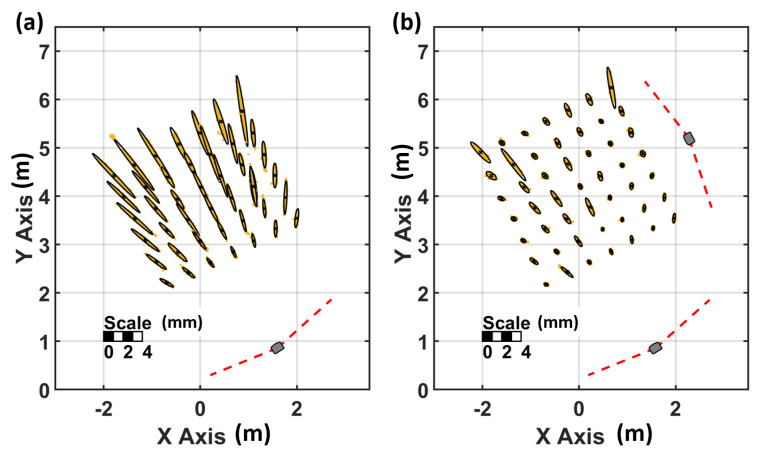
Noise pattern of the Tracker with one (**a**) and two lighthouses (**b**).

**Figure 6:**
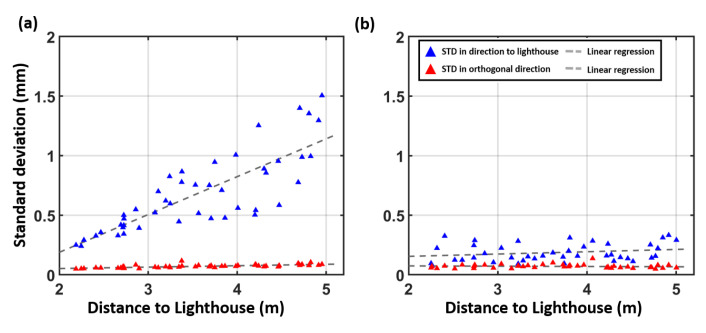
Distance dependency of the noise patterns of the tracker positions (**a**) with one lighthouse and (**b**) with two lighthouses.

To investigate the impact on the Tracker’s precision, 500 static measurements have been carried out in a setup with one lighthouse and in a setup with two lighthouses. To ensure good spatial distribution, a grid has been measured with 0.5 m spacing. The distribution of the grid points and the lighthouse configuration can be seen in Figure 5. In Figure 5a, the noise pattern is orientated in the direction of the single lighthouse and it can be seen that the magnitude increases with the distance to the lighthouse. Figure 6a shows a detailed inspection of the noise pattern when a single lighthouse is used.

The corresponding numerical values are displayed in Table 3. The linear regression of the deviation in direction to the lighthouse shows a significant slope of 0.32 mm per metre (precision of the slope 0.04 mm/m) with the use of a single lighthouse. The further the Tracker is away from the single lighthouse, the higher the noise in the direction to the lighthouse.

In Figure 5b, the use of two lighthouses reduces the measurement noise in direction to the lighthouse significantly. The majority of the grid points show an nearly isotropic noise distribution. In comparison to Figure 6a, no significant distance dependence of the noise (see Table 3, second row) can be observed in Figure 6b. Although two identical lighthouses have been used, some of the ellipses in Figure 5b are still headed towards a specific lighthouse. This behaviour indicates that the algorithm of SteamVR chooses one of the used lighthouses to be a “master” lighthouse with higher influence on the position estimation.

## 6. Accuracy of the VR System

The absolute deviation of a measurement from a true value is called accuracy. The investigation of this parameter requires external information as the true value is unknown in most cases. A reference value is often used, which is determined by a sensor which has a measurement accuracy of a higher order.

### 6.1. Orientation of the Reference Plane

A major issue that was revealed by Niehorster [4] for the lighthouse system of the first generation was a tilt in the horizontal reference plane that appeared to be very unstable.

To investigate this effect for the Lighthouse system 2.0 the experiment of Section 5.2 has been repeated with external information by a calibrated geodetic total station (Leica Viva TS15, Leica Geosystems AG, Heerbrugg, Switzerland) which provided ground truth.

**Figure 7:**
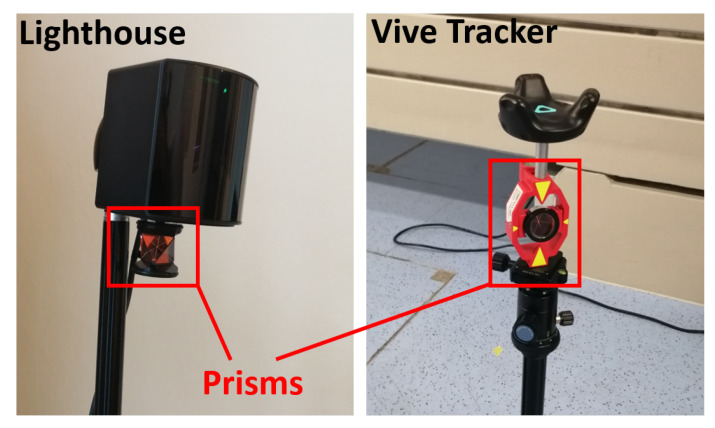
Mounted surveying prisms on the lighthouse and tracker.

As it can be seen in Figure 7, the used lighthouses and the Vive Tracker have been equipped with surveying prisms. The 3D positions of these prisms have been tracked with millimetre accuracy by the total station throughout the whole experiment. The 3D coordinates are measured in a local coordinate system. Due to the internal compensator of the total station, the horizontal plane of this local coordinate system is levelled with an accuracy of 0.5″ (0.00014°) [25]. For comparison, the Vive Tracker measurements have been transformed with a 2D Helmert transformation onto the corresponding total station measurements.

**Figure 8:**
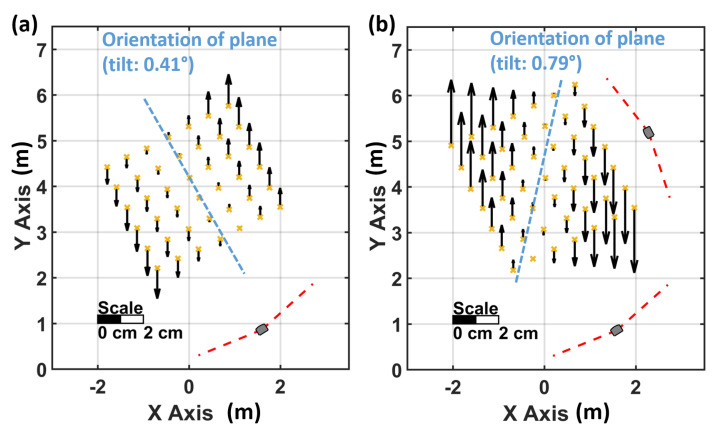
Height deviations to total station measurements with (**a**) one and (**b**) two lighthouses.

The height deviations in Figure 8 show a systematic behaviour and prove a tilted reference plane also for the lighthouse system of the second generation. A best fitting reference plane in Figure 8a has a centreline that is orientated towards the single lighthouse and has a calculated inclination of 0.416${}^{\circ }$ (precision 0.005${}^{\circ }$). Resetting the VR system with a new room setup did not improve the vertical tilt of the plane (tilt of first room setup: 0.416${}^{\circ }$, tilt of second room setup: 0.423${}^{\circ }$). When the HMD faces tracking issues and regains tracking, the whole lighthouse system is recalculated, also in this case no significant improvement of the tilt has been observed (new tilt: 0.405${}^{\circ }$).

A significant effect on the reference plane can be observed in a setup with two lighthouses. Figure 8b shows that both lighthouses contribute to the reference plane, therefore the rotation axis of the plane is aligned between the two lighthouses and a tilt of this plane of 0.788${}^{\circ }$ (precision 0.005${}^{\circ }$) was derived. When the HMD faces tracking issues in this setup, the “master” lighthouse can change due to internal quality parameters. If this happens, the alignment of the reference plane changes with regard to the new “master” lighthouse.

### 6.2. Lighthouse Behaviour and 2D Accuracy

As displayed in Figure 7, a surveying prism has also been mounted underneath each lighthouse to investigate the behaviour of the lighthouse coordinates as well. Due to the known orientation of the lighthouses and the offset between the centre point and the camera screw thread (can be obtained from the model that is provided by the SteamVR plugin in the game engine Unity), the coordinates of the prism centre can be calculated in the local lighthouse coordinate system. These local prism coordinates have been transformed via the same 2D Helmert transformation, which has been obtained from the Vive Tracker positions and the total station measurements. With this procedure the relative accuracy of all the Tracker measurements and the relative accuracy between the Tracker measurements and the corresponding lighthouse positions can be investigated.

**Figure 9:**
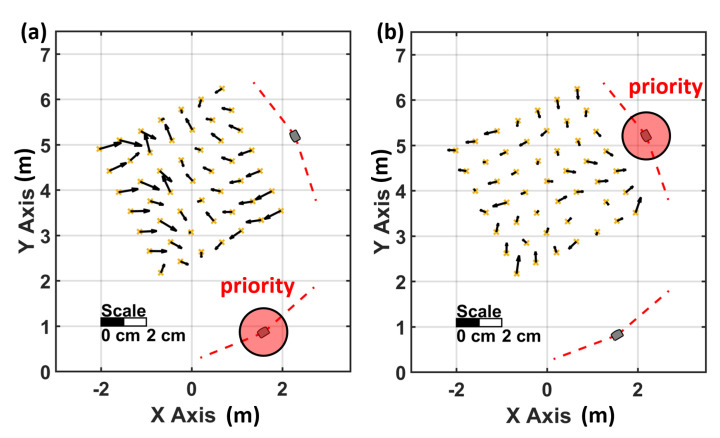
Horizontal deviations to total station measurements (**a**) before and (**b**) after bad tracking issues.

Figure 9 provides the horizontal deviations of the Vive Tracker measurements for the same setup, which can be seen in Figure 5b and Figure 8b. Figure 9b shows the results for the same setup after the HMD faced bad tracking issues and the lighthouse system was recalculated. The bad tracking issues have been caused intentionally by obstructing the lines of sight between the HMD and the lighthouses. A detailed inspection of the corresponding noise pattern proved that the “master” lighthouse had changed.

**Figure 10:**
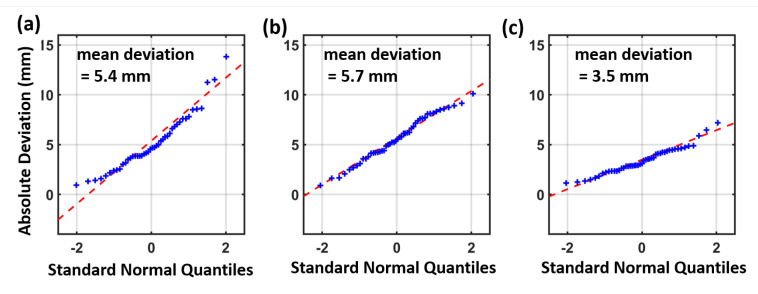
QQ-Plot with horizontal (**a**) with a single lighthouse, (**b**) with two lighthouses, and (**c**) with two lighthouses after bad tracking issues.

The deviations that are displayed in Figure 9a and Figure 10b are between 1 and 12 mm. The calculated position for the “master” lighthouse has a total deviation of 10 mm to the total station measurements. The second lighthouse has a position error of 100 mm relative to the Vive Tracker measurements. After a recalculation of the lighthouse system in Figure 9b, the priority of the lighthouses has changed. The new “master” lighthouse matches the reference coordinates within 2 mm. The former “master” lighthouse has now a position error of 55 mm, which is an overall improvement compared to the configuration in Figure 9a. The Vive Tracker measurements have now a mean difference of 3.5 mm to the total station measurements in this setup, the corresponding QQ-Plot is displayed in Figure 10c. On the basis of these results it can be said that the coordinates of the “master” lighthouse fit best to the Vive Tracker measurements and the other lighthouses are prone to vast position errors.

Experiments with the same setup and the use of a single lighthouse (Figure 10a) have proven that the use of two lighthouses (Figure 10c) can lead to a significant accuracy improvement. The mean difference of 5.4 mm in a setup with a single lighthouse can be reduced to a mean difference of 3.5 mm with the use of two lighthouses. However in the setup with two lighthouses, before the recalculation (seen in Figure 10b), a comparable result has been achieved to the use of a single lighthouse.

The observed lighthouse behaviour indicates that the system is designed to adapt on the fly. The central element of the SteamVR algorithm is the HMD which defines the “master” lighthouse and calculates the position for all the other visible lighthouses. Due to internal quality parameters the HMD can choose the best available lighthouse to be the new “master” lighthouse and defines its coordinates as stable. The coordinates of all visible lighthouses are then altered on the basis of the new “master” lighthouse. The experiment in Figure 9 has shown that this estimation of the lighthouse coordinates can have tensions of several centimetres, which affects the position estimation of all other devices, excluding the HMD.

### 6.3. Deviations from a Reference Path

**Figure 11:**
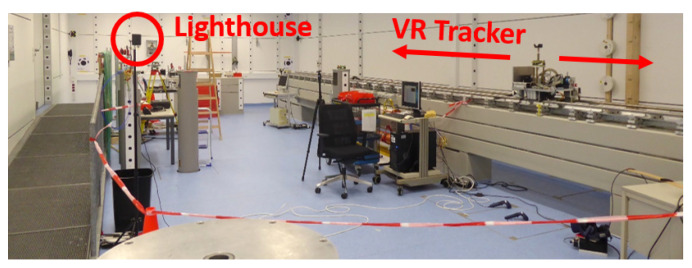
Setup on the horizontal comparator with one lighthouse.

For the investigation of the performance of the Vive Tracker for the whole working range of a lighthouse the device has been mounted on the horizontal comparator of the measurement laboratory. To achieve a representative result by avoiding a changing “master” lighthouse, only a single lighthouse has been used in this setup (Figure 11).

The VR Tracker has been moved along the rail in 25 cm steps. At each stop, 500 static measurements have been recorded. The mean values of these measurements can be compared to the interferometer measurements. In this setup, several tests have been carried out with varying distances between lighthouse and VR tracker. These tests have confirmed that the working range of the lighthouses lasts up to 7 m.

**Figure 12:**
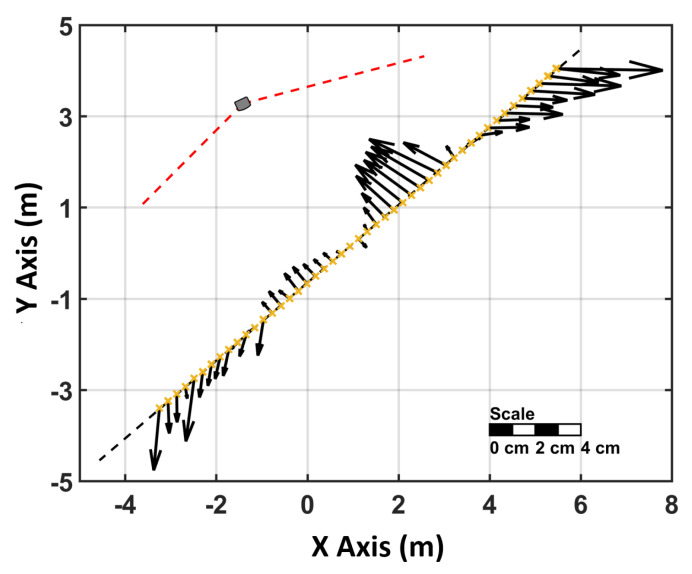
Horizontal deviations between the VR Tracker and the laser interferometer of the comparator.

**Figure 13:**
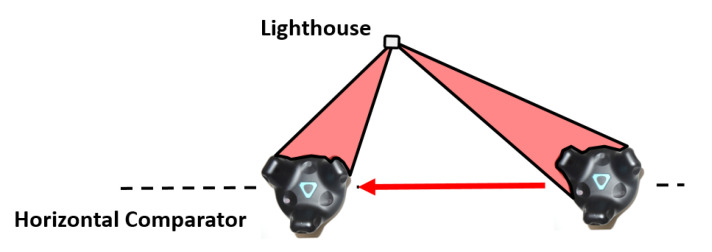
Visibility of different IR diodes as the tracker moves along the horizontal comparator.

The deviations between the VR Tracker and the true positions on the comparator are displayed in Figure 12 and can reach up to 40 mm. The deviations are headed towards the lighthouse like the noise pattern. All experiments on the comparator have been repeated twice and the deviations have been reproducible within 3 mm. Regarding the systematic effect of the deviations, which can be seen in Figure 12, it has to be noted that the orientation of the tracker with respect to the lighthouse changes as the tracker moves along the horizontal comparator. It was assumed that this effect occurs due to the change of the visibility of the IR diodes on the VR Tracker during the experiment (Figure 13).

### 6.4. Rotation Dependency in a Setup with a Single Lighthouse

**Figure 14:**
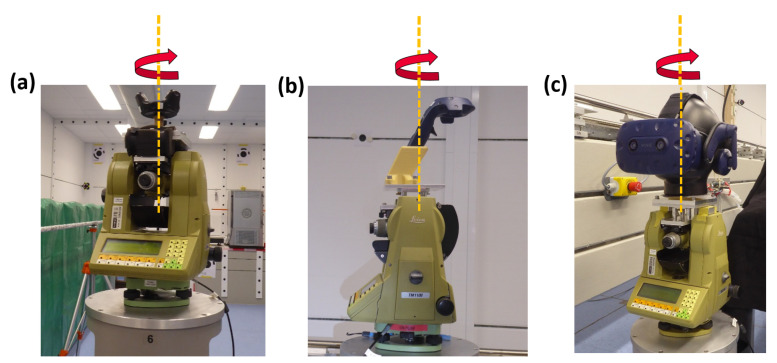
Setup on the TM1100 rotation platform: (**a**) for the tracker, (**b**) for the controllers and (**c**) for the HMD.

To investigate the contribution of different IR diodes to the position estimation, all VR devices have been mounted onto a rotation device. The rotation device was a Leica TM1100 total station (Leica Geosystems AG, Heerbrugg, Switzerland) [26], which rotated automatically 360${}^{\circ }$ with a 1${}^{\circ }$ step-width. At each step, 500 positions and orientations have been recorded. For each rotation step the mean value of the position and the corresponding standard deviations have been computed. As seen in Figure 14, the controller and the HMD have been mounted eccentrically; this has been corrected numerically by circle fits in the data analysis.

**Figure 15:**
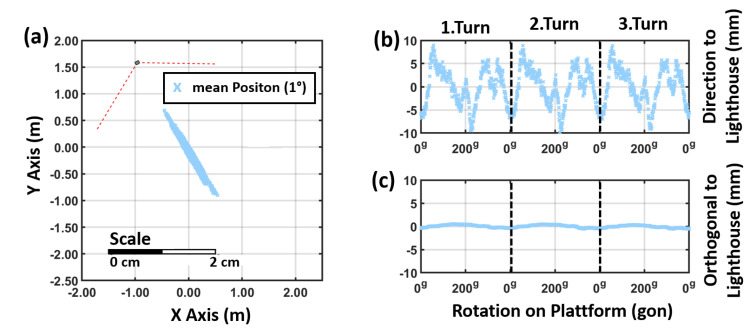
Horizontal position variation a VR Tracker with a single lighthouse: (**a**) horizontal plot, (**b**) in direction to the lighthouse and (**c**) in orthogonal direction. (400 g = 360${}^{\circ }$)

The elliptic-shaped noise pattern of the 500 measurements can be observed at each 1${}^{\circ }$ rotation step. Moreover, during the rotation the mean position of tracker (calculated out of the 500 measurements at each step) faces a significant movement. Figure 15a shows the results for the rotation for a VR Tracker in a setup with one lighthouse which was 2 m away. The deviations of the mean positions are orientated in direction to the lighthouse which correlates with the noise pattern and the deviation shown in Figure 3. Each rotation has been carried out three times and the results are reproducible within a few millimetres (see Figure 15b,c).

**Figure 16:**
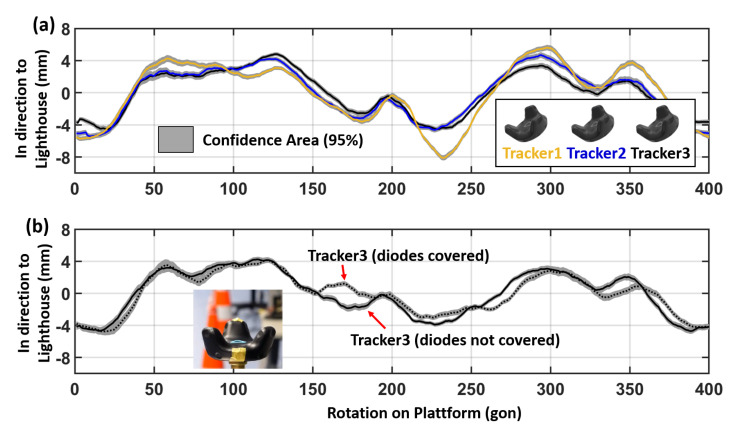
Deviation of the mean positions during the rotation test (**a**) with three different VR Trackers and (**b**) with covered IR diodes. Distance to lighthouse: 2 m.

Several rotation tests in different setups (variation in tilt and height of the lighthouse and distance to the Tracker) have shown that a systematic pattern is inherent in all measurements. The behaviour of the systematic pattern and the magnitude changes in different setups due to the visible IR diodes on the Tracker. To see if other VR Trackers are affected by these systematic deviations as well, three different VR Trackers have been rotated in exactly the same setup in 2 m distance. The results can be seen in Figure 16.

Plotted are the mean deviations of the 500 individual measurements at each orientation and as a grey band the 95% confidence area. The overall systematic with the same magnitude appears in all datasets (see Figure 16a), although each VR Tracker shows locally significant differences. Comparing the three Trackers it can be seen that Tracker2 and Tracker3 fit better to each other than Tracker1. This may indicate a different quality in the IR diodes of the VR Trackers. To simulate a malfunction of IR diodes the experiment has been repeated with covered diodes on the VR Tracker3. This has led to similar local deviations from the overall systematic displayed in Figure 16b.

**Figure 17:**
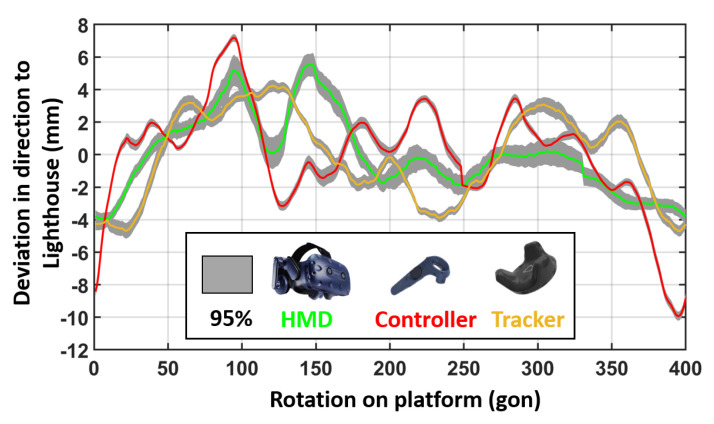
Horizontal position variation in direction to the lighthouse for different VR devices. Distance to lighthouse: 2 m.

To investigate this effect on other Vive devices, rotation tests with the HMD and a controller have been carried out as well. In Figure 17, the rotation results for different VR devices are shown in a comparable measurement configuration. It can be seen that all devices face a systematic rotation behaviour of several millimetres up to one centimetre. Due to the arrangements of the IR diodes on the devices these systematic patterns differ significantly from each other.

### 6.5. Rotation Dependency in a Setup with Two Lighthouses

**Figure 18:**
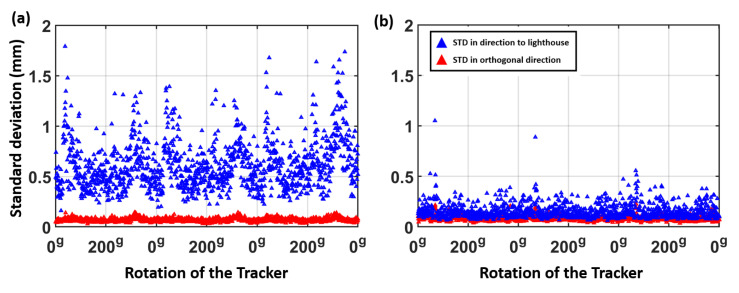
Investigation of the noise pattern at each rotation step in a setup (**a**) with one lighthouse and (**b**) with two lighthouses.

The rotation test with the VR Tracker has been repeated in a setup with two lighthouses. Figure 18 shows a detailed inspection of the noise pattern at each rotation step. In Figure 18a, a higher standard deviation in direction to the lighthouse can be seen. Due to the use of two lighthouses in Figure 18b the noise in direction to the “master” lighthouse has been reduced and a nearly isotropic noise pattern of the 500 measurements can be observed at each rotation step.

**Figure 19:**
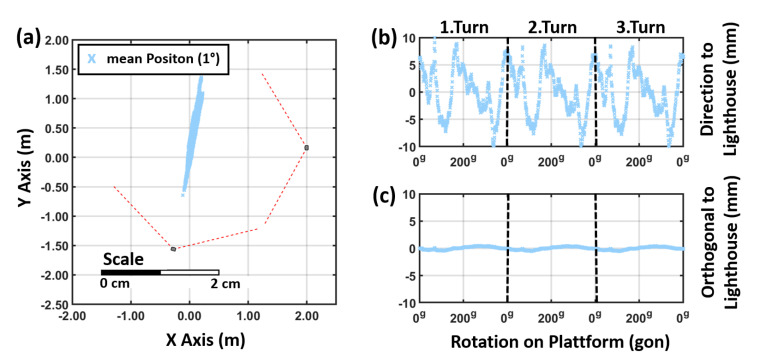
Results of the rotation test for a VR Tracker with two lighthouses (**a**) horizontal plot, (**b**) in direction to the lighthouse and (**c**) in orthogonal direction.

However, the mean positions at each rotation step show the same systematic deviation in Figure 19, as it can be seen in Figure 15 in a setup with only one lighthouse. The rotation systematic is clearly orientated towards in direction to the “master” lighthouse in the setup with two lighthouses. This means that the use of two lighthouses affects the noise distribution, but has no affect on the absolute deviations due to the rotation of the devices.

## 7. Discussion

The system investigations on the VR system HTC Vive Pro have shown that the Lighthouse system 2.0 is capable of reaching a position accuracy of a few millimetres with the Vive Tracker device. This is a factor of 10 worse than the reported accuracy of the iGPS system.

However, a big issue is that the position estimation by SteamVR is a black box. The designed workflow aims for a quick setup, needs no information from outside the system and has a big focus on continuity, which is needed for the well-being of the user [27].

Regarding the experiments carried out in the measurement laboratory, it can be said that the position estimation of the used lighthouses can have discrepancies of a few centimetres and the whole system is dependent on the current lighthouse with the highest priority. When moving from one lighthouse to another lighthouse, the system can decide to re-adjust all visible lighthouses. On the one hand, this behaviour makes the system hard to control in mixed reality applications, and, on the other hand, this can lead to problems when using multiple lighthouses in a row to extend the working range. Valve’s SteamVR Software supports the use of up to 16 lighthouses in a single setup. Therefore, the working range of the lighthouse system can be expanded throughout several rooms. Moving with the HMD from one room to another room will force the lighthouse system to recalculate the lighthouse priorities and will affect the overall accuracy. Investigations of the system’s behaviour with an increased number of lighthouses will be part of future research activities.

A strategy for improving the accuracy of the VR system would be the use the lighthouse system in a fixed setup with known positions of the lighthouses. This would decrease the active role of the HMD in the position estimation and the system would have enormous potential in various industrial applications. However, this would mean a major alteration in the SteamVR tracking approach.

Another approach for improving the accuracy of the VR system could be the use of several Vive Trackers on known positions in the room. The Vive Tracker has shown a high relative accuracy in the surrounding area, and it is affected by all systematic changes of the lighthouse system. Equipping areas which have a high need for spatial accuracy with at least three Vive Trackers, observing their behaviour and applying the changes to the used models in VR applications could sustain a high relative accuracy, which might be sufficient for most mixed reality applications.

Investigations by the University of British Columbia showed that the accuracy of another state of the art VR system, the Oculus Touch, is similar under optimal conditions [28]. An accuracy of a few millimetres was reported; however, the orientation of the controller has been constant throughout the whole experiment and the measurement system, which was used as reference, was only capable of 1D movements.

## 8. Conclusions

The behaviour of the position of the Vive HTC Pro system showed a high reproducibility of a few millimetres in all experiments. The major impacts on the overall accuracy have been systematic effects like a tilted reference plane, a poor calibration of the VR devices and a changing hierarchy in the lighthouse system. These effects can lead to position errors of more than one centimetre. Regarding the rotation systematics of the VR devices, they have shown a high dependency on the setup and the visibility of the IR diodes by the lighthouse. However, the tests have proven that these systematics show a high reproducibility in a fixed setup. Therefore, calibration functions can be derived and when using the VR Tracker in a fixed setup (e.g., mounted on a driving wheel) it should be possible to numerically correct these rotation systematics.

Furthermore, it has been proven, that also the second generation of the HTC Pro system does not provide a horizontal reference plane. In our experiments, an inclination of about 0.4${}^{\circ }$ with a single lighthouse and 0.8${}^{\circ }$ with two lighthouses have been observed.

## Figures and Tables

**Table 1:** List with acronyms and notations.

Acronyms or Notations	Meaning
pose	3D position and orientation
significant	based on a statistical test with *p* = 0.95
noise or precision	standard deviation (1 σ)
HMD	Head Mounted Display
VR	Virtual Reality
QQ-Plot	Quantile Quantile Plot
IR	Infrared
HTC	High Tech Computer Corporation
400 Gon (g)	360 Degrees (${}^{\circ }$)

**Table 2:** Numerical values for the noise distribution of different devices.

Device Typ	Amount of Diodes	σ Orthogonal to Lighthouse	σ in Direction to Lighthouse
HMD	31	0.1 mm	0.3 mm
Controller	24	0.2 mm	0.4 mm
Tracker	22	0.3 mm	0.5 mm

**Table 3:** Numerical values of precision investigation with regard to the lighthouse and significance (Sig.) at a confidence level of 95%.

	Orthogonal to Lighthouse			in Direction to Lighthouse		
**Lighthouses**	**Slope**	**σ Slope**	**Sig.**	**Slope**	**σ Slope**	**Sig.**
1	0.012 mm	0.002 mm	Yes	0.318 mm	0.039 mm	Yes
2	0.002 mm	0.002 mm	No	0.019 mm	0.011 mm	No

## Data Availability

Not applicable.
